# Electrospun antimicrobial poly(lactic acid) foams with nanocellulose for enhanced hydrophilicity and controlled drug release

**DOI:** 10.1039/d4ra08580a

**Published:** 2025-02-28

**Authors:** Daira Sleinus, María José Lovato, Oskars Platnieks, Alisa Sabalina, Sergejs Gaidukovs, Lourdes Franco, Jordi Puiggalí, Luis J. del Valle

**Affiliations:** a Institute of Chemistry and Chemical Technology, Faculty of Natural Sciences and Technology, Riga Technical University P. Valdena 3, LV-1048 Riga Latvia sergejs.gaidukovs@rtu.lv; b Departament d'Enginyeria Química, Escola d'Enginyeria de Barcelona Est (EEBE), Universitat Politècnica de Catalunya – Barcelona Tech (UPC, ) Av. Eduard Maristany 10–14 Barcelona 08019 Spain luis.javier.del.valle@upc.edu; c Barcelona Research Centre in Multiscale Science and Engineering, Universitat Politècnica de Catalunya, BarcelonaTech (UPC) Av. Eduard Maristany, 16 Barcelona 08019 Spain

## Abstract

This study explores an advanced approach to enhancing the antimicrobial efficacy and hydrophilicity of poly(lactic acid) (PLA) scaffolds through the strategic incorporation of cellulose nanocrystals (CNC). The compatibility between these biodegradable polymers was investigated to optimize antimicrobial agent release while preserving structural integrity. PLA nanocomposites incorporating the antimicrobial agents curcumin (Cur) or polyhexamethylene biguanide (PHMB) were fabricated using three distinct electrospinning-based methodologies. The antibacterial properties were assessed *via* a disc diffusion test against five bacterial strains: *Escherichia coli*, *Escherichia coli* B+, *Lactobacillus salivarius*, *Streptococcus sanguinis*, and *Streptococcus mutans*. In addition, drug release experiments were conducted to determine the diffusion kinetics in a simulated blood serum medium, demonstrating sustained drug release for up to 98 hours. PHMB demonstrated potent antibacterial activity, while curcumin primarily exhibited bacteriostatic effects. The thermal stability of the nanocomposites exhibited an increase of up to 41 °C in the maximum degradation temperature. The mechanical properties were assessed to further examine the interactions between CNC and PLA and the possibility to reshape the materials for different delivery approaches. The findings underscore the crucial role of CNC in modulating the interaction between PLA and antimicrobial agents, making it a promising candidate for biomedical applications requiring controlled drug release. This study provides valuable insights into the structural, thermal, and antibacterial performance of CNC–PLA nanocomposites, establishing a strong foundation for the development of advanced biodegradable materials for drug delivery and antimicrobial applications.

## Abstract 2

This study explores an advanced approach to enhancing the antimicrobial efficacy and drug release of poly(lactic acid) (PLA) scaffolds through the strategic incorporation of cellulose nanocrystals (CNC).

## Graphical abstract

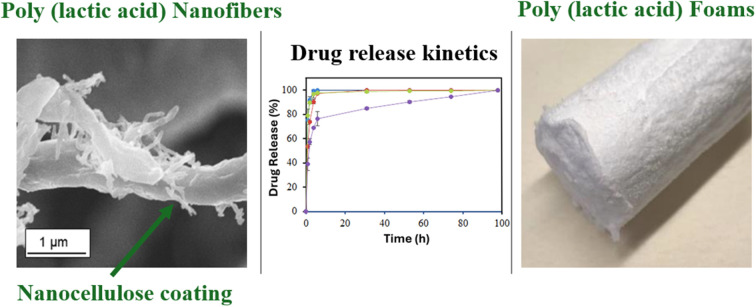

## 1. Introduction

In recent years, biodegradable materials have garnered considerable interest due to the growing awareness of environmental issues. Among these materials, bio-based aliphatic polyesters are prominent, known for their biodegradability, biocompatibility, as well as their non-toxic characteristics.^1,2^ These features make them apt for a range of contemporary applications, including food packaging, biomedical supports and other disposables.^3,4^ Specifically, poly(lactic acid) (PLA) not only offers these advantages but also competes effectively with fossil-based alternatives in terms of cost and mechanical properties.^5^ According to recent market analyses, PLA is increasingly favored in various applications due to its lower environmental impact and comparable performance metrics.^6^

Electrospinning has emerged as a versatile technique for crafting nano- and microfibrous scaffolds, providing a high surface area-to-volume ratio and adjustable porosity, which are critical features for innovative uses. However, the application of PLA-based materials in the biomedical sector faces challenges, particularly in augmenting their antimicrobial efficacy and hydrophilicity.^7^ These enhancements are essential to facilitate cellular adhesion and proliferation.^8^ Studies have shown that modifying the surface properties of PLA can significantly improve its interaction with biological tissues.^8^ Furthermore, the integration of antimicrobial functionality into PLA fibers extends their utility beyond the biomedical sphere.^9^ Such functionalization enables the preparation of films, mats, membranes, *etc.*, that are not only useful in food packaging but also crucial in water purification processes.^10^ Moreover, adding nanoparticles can enhance the properties of electrospun fibers relative to neat PLA fibers.^11^ Cellulose nanocrystals (CNC) have revived attention due to their intrinsic properties, such as surface area, hydrophilic affinity, nanoscale dimension, excellent flexural rigidity and mechanical properties, and biodegradability.^12,13^

The incorporation of nanocellulose into PLA to enhance the structural, mechanical, and thermal properties of electrospun fibers has been well reported in the literature.^14–16^ However, research on the strategic integration of CNC into PLA nanofibers to improve drug release kinetics and antimicrobial activity remains relatively limited. Zhou *et al.* developed electrospun bio-nanocomposite scaffolds using maleic anhydride (MAH)-grafted PLA reinforced with CNC.^17^ The addition of CNCs significantly improved mechanical properties, thermal stability, and degradation resistance, while surface grafting with MAH enhanced interfacial adhesion between CNCs and PLA, leading to a more uniform and finer fiber structure. The optimized scaffold with 5 wt% CNCs exhibited superior tensile strength, controlled degradation, and high biocompatibility with human adipose-derived mesenchymal stem cells (hASCs). Wu *et al.* investigated the use of CNC and polyethylene glycol (PEG) as bifunctional reinforcing and compatibilizing agents in electrospun PLA nanofibers for controlled long-term drug release.^18^ The addition of CNC/PEG improved the nanofibers' mechanical properties, hydrophilicity, and thermal stability while enabling high drug loading efficiency (up to 98%) and sustained drug release over 1032 hours. Cheng *et al.* reported electrospun poly(3-hydroxybutyrate-*co*-3-hydroxyvalerate) (PHBV) nanofibrous membranes for biomedical applications by incorporating CNC.^19^ The addition of CNCs significantly improved the membranes' mechanical strength, thermal stability, and hydrophilicity, leading to enhanced cytocompatibility and prolonged drug release. The optimized membranes demonstrated sustained drug release for over 540 hours. Salmani *et al.* prepared PLA and poly(ε-caprolactone) (PCL) macroporous scaffolds for bone tissue engineering by incorporating CNC and a PCL–PEG–PCL triblock copolymer.^20^ The CNC acts as a stabilizer, preventing PCL droplet coalescence, while the triblock copolymer improves miscibility between PLA and PCL by reducing interfacial tension, leading to better pore uniformity, increased water absorption, and improved mechanical stability. The optimized scaffold containing 10% triblock copolymer and 1.0% CNC exhibited high biocompatibility, enhanced osteogenic differentiation of human mesenchymal stem cells (hMSCs), and the highest calcium deposition, making it a promising material for bone regeneration applications. Mohammadalinejhad *et al.* reported PLA nanocomposite films reinforced with silver nanoparticle (AgNP)-decorated cellulose-based nanofibers to improve their mechanical strength, barrier properties, and antimicrobial effectiveness for active food packaging applications.^21^ The study evaluated three different nanofibers: cellulose nanofibers (CNF), chitosan nanofibers (CHNF), and lignocellulose nanofibers (LCNF). Among these, LCNF was found to be the most compatible with the PLA matrix, leading to superior mechanical stability, reduced water vapor permeability, and controlled release of AgNPs, which prolonged the antimicrobial effect. However, CNF-AgNPs tended to aggregate within the PLA matrix, reducing their overall effectiveness.

We developed and systematically compared three distinct fabrication methods for incorporating antimicrobial agents—curcumin (Cur) and polyhexamethylene biguanide (PHMB)—within the electrospun PLA materials. The incorporation of CNC was strategically designed to facilitate controlled drug release and optimize antibacterial performance. This work supplements previous studies by further exploring compatibility and interactions of CNC and PLA as nanofiber structure-forming materials. We show CNC's pivotal role in modulating the diffusion of active compounds. Moreover, drug release experiments confirm that CNC promotes rapid diffusion, particularly when applied as a coating. In addition, the research provides a comprehensive structural, thermal, and mechanical characterization of the developed systems. The findings lay a strong foundation for further advancements in bioactive PLA-based materials and open new avenues for their practical implementation in antimicrobial disposables, wound dressings, and healthcare applications.

## 2. Materials and methods

### 2.1. Materials

Qualitative filter paper with low ash grade was obtained from Scharlau (Scharlab, Spain) and used as source for CNC. Polylactic acid (PLA) with grade Ingeo™ 2002D (NatureWorks LLC) was selected as polymer matrix. The selected PLA grade is amorphous and is designed for extrusion and thermoforming processing, aimed at potential applications in dairy containers, utensils, transparent food containers, blister packaging, and cold drink cups. Chemical reagents were secured from various suppliers' sulfuric acid (95–98%) from Honeywell, acetone (synthesis grade) was obtained from Sigma Aldrich, chloroform (99.9%, anhydrous) from Scharlau (Scharlab, Spain). Curcumin (Cur) was purchased from Sigma-Aldrich. Polyhexamethylene biguanide (PHMB) was purchased from Sharon Laboratories Ltd, Luria–Bertani (LB) broth from Fisher, and Bacto Agar from Becton Dickinson. Nalidixic acid (NA) 30 μg susceptibility test discs were purchased from Fisher Scientific.

### 2.2. CNC preparation

**Fig. 1:**
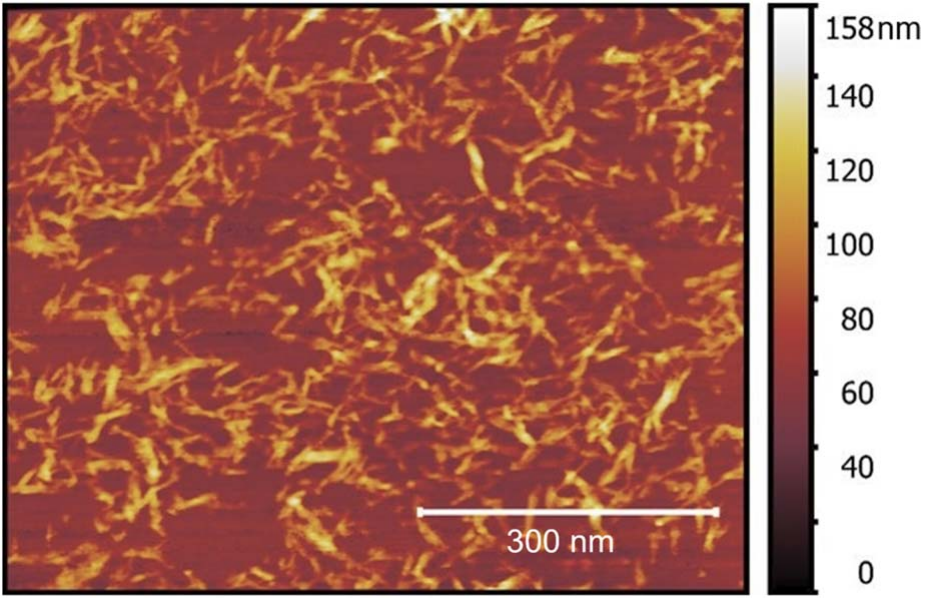
AFM topography image of CNC.

10 g of shredded filter paper was mixed with 91 ml of 66% w/w sulfuric acid water solution using a magnetic stirrer at temperature of 25 °C. After 3 hours, 187 ml of 50% w/w sulfuric acid water solution was added and stirred for few minutes to homogenize the mixture. Immediately afterward, the suspension was subjected to a first stage of centrifugation at 23 °C using a Sorvall RC 5B Plus centrifuge for ten minutes at 5000 rpm. The CNC layer was separated and diluted with chilled water to approximately four times its initial volume and left for 24 hours. Excess water was decanted followed by a second centrifugation stage consisting of three processes at 5000 rpm for 10 min each (23 °C). The CNC layer was collected at the end of centrifugation and dialyzed for two weeks until the pH was neutralized. After dialysis, the wt% of CNC in the suspension was calculated by taking a known, small amount of the suspension and determining the CNC mass after oven and vacuum drying. Fig. 1 shows the prepared CNC AFM topography, indicating that the CNC particle diameter is 13 ± 8 nm and the length is 235 ± 71 nm.

### 2.3. Preparation of PLA fibers by electrospinning method

4 g of PLA were dissolved in 40 mL 25% v/v acetone/chloroform solution. This solution was used for reference PLA fiber preparation. Two solutions with antimicrobial agent were prepared (curcumin or PHMB). The concentration of antimicrobial agents was 1% w/w relative to PLA. The solutions were electrospun to prepare PLA fibers using a GENEQ Inc. device. The solution was placed in a 10 ml syringe, with the needle tip (inner diameter = 0.84 mm) positioned 15.0 cm from a foil-covered surface used as a collector. The applied electric voltage was 15 kV. The feed rate was fixed at 10 ml h^−1^.

### 2.4. Foam and film preparation

Electrospun fiber membranes were cut into smaller pieces and dispersed in water (ratio 1 : 20 w/w) using T 18 Ultra-Turrax® (IKA) set at 10 000 rpm for 15 min. Afterwards, CNC water suspension (1.78 wt%) was added to PLA fiber suspension and the weight ratio 1 : 100 (CNC : PLA), with exception of PLA_2CNC, which used 2 : 100 ratio. In addition, some samples were coated with mix of CNC and antimicrobial agents (Cur or PHMB) with fixed mass 1% w/w relative to PLA. After repeated homogenization, the suspension was freeze-dried using LyoQuest (Telsar) device. The freeze-drying yielded sample foams (Fig. 3a).

**Fig. 2:**
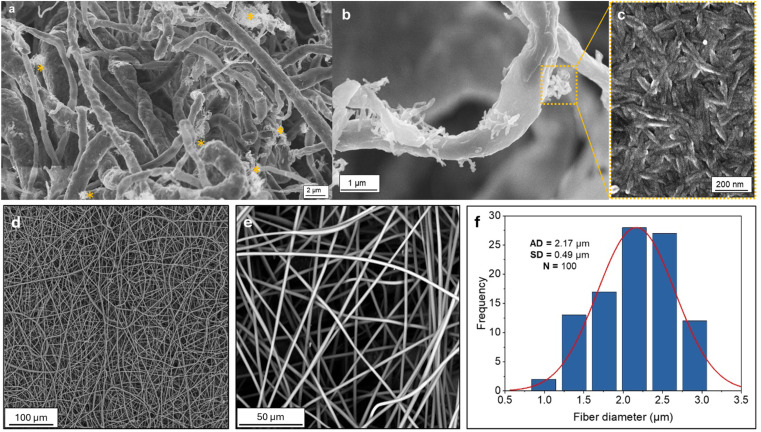
SEM micrographs of electrospun PLA fibers: (a) PLA fibers coated with CNC at a 10 : 100 (CNC : PLA) weight ratio; (b) higher magnification of a single coated PLA fiber, with a yellow inset highlighting a CNC bundle; (c) further enlargement of the highlighted region, revealing the CNC structure; (d) pure electrospun PLA fibers and (e) their magnification; (f) diameter distribution histogram, where AD denotes the average fiber diameter, SD the standard deviation, and *N* the number of fibers measured. Asterisks (*) in (a) mark regions with notable CNC concentrations.

In addition, a composition with a 10 : 100 weight ratio (CNC : PLA) was prepared to illustrate the CNC coating on the nanofiber surfaces, as shown in Fig. 2a–c. The size of pure PLA nanofibers was examined by SEM (Fig. 2d and e), and the corresponding histogram is presented in Fig. 2f. The mean diameter of the PLA fibers which was estimated by averaging the measurements of 100 fibers from the mat ranges from approximately 2.17 ± 0.5 μm.

**Fig. 3:**
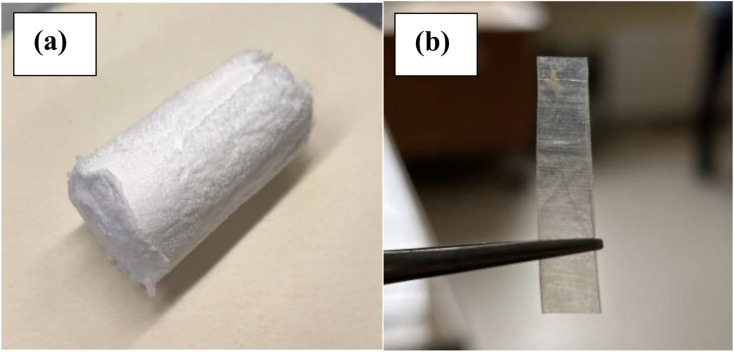
Samples (a) PLA(C)Cur and (b) PLA_CNC.

Selected samples were covered with Teflon films and aluminum foils and compression molded between two hot plates for 30 s at 160 °C. Resultant pressed films had a thickness of around 0.15 mm (Fig. 3b). Full list of samples is presented in Table 1 and the concentration of antimicrobial agents were 1% w/w relative to PLA, independent of addition method (in the fibers or as coating).

**Table 1:** Samples abbreviations and composition

Sample abbreviation	Form	Fiber compositions	Fiber coating
PLA	Film	PLA	—
PLA_CNC	Film	PLA	CNC
PLA_2CNC	Film	PLA	2 × CNC
PLA(C)	Foam	PLA	CNC
PLA_Cur	Foam	PLA + Cur	CNC
PLA_PHMB	Foam	PLA + PHMB	CNC
PLA(C)Cur	Foam	PLA	CNC + Cur
PLA(C)PHMB	Foam	PLA	CNC + PHMB

### 2.5 Methods

Atomic force microscopy (AFM) topography measurements were obtained with Smena by NT-MDT (Russia). The samples were prepared on the silicon substrate and tested in the semicontact mode (tapping mode) using HA_NC (ETALON) tip. The samples were produced by evaporating water from droplet of CNC suspension with a concentration of 0.002 wt%. The diameter was derived from topographic height measurements, enabling the determination of a correction factor to account for the apparent width introduced by the measurement tip. This correction was subsequently applied to the length measurements.

Scanning electron microscopy (SEM) was employed to examine the morphology of samples. Micrographs were obtained with a Phenom XL Desktop SEM instrument. Samples were mounted on a double-sided adhesive carbon disc and were sputter-coated with a thin layer of carbon to prevent sample charging problems using a K950X Turbo Evaporator.

A DSC-1 (Mettler Toledo) analyzer was used to perform differential scanning calorimetry (DSC) analysis on the samples. Under nitrogen purge, samples in aluminum pans weighing about 10 mg were heated to 200 °C, held there for 5 minutes, cooled to 25 °C, held there for 5 minutes, and then heated to 200 °C once more. The heating/cooling rate was constant 10 °C min^−1^.

Fourier transform infrared spectroscopy (FTIR) in attenuated total reflectance mode was used for the sample investigation with a Nicolet 6700 (Thermo Scientific, Germany) device. A resolution of 4 cm^−1^ in the 800–4000 cm^−1^ region was used to perform sixteen measurements with a measurement error of 1%. The average spectrum is displayed.

The thermal stability was examined using thermogravimetric analysis (TGA) with TG50 equipment (Mettler Toledo) in accordance with ASTM D3850 standard. The samples were heated at a rate of 10 °C min^−1^ in an air atmosphere between 25 and 700 °C.

Dynamic mechanical analysis (DMA) was performed in a tension mode using a DMA/SDTA861e (Mettler Toledo). Rectangular samples (8.5 × 4.0 × 0.15 mm) were preconditioned in 40% relative humidity (RH) at room temperature (22 °C) for 24 h. The experiment used a temperature range of −70 to +100 °C, 5 N of applied force, an elongation of 10 μm, 1 Hz frequency, and a heating rate of 3 °C min^−1^.

A Tinius Olsen type 25ST (USA) universal testing machine was used to measure the tensile characteristics. The preconditioning of rectangular samples (5.0 × 1.0 × 0.015 cm) was done in the same way as for DMA. A 5 kN load cell was employed, and the testing crosshead speed was set at 1.0 mm min^−1^. For every sample, ten measurements were carried out under ambient conditions (22 °C, RH 40%).

Antimicrobial activity assay was performed with disc diffusion test over LB agar, set up with five bacteria usually used in laboratory settings, such as *Escherichia coli* biofilm-negative CECT 101 (*E. coli*), *Escherichia coli* biofilm-positive (B+) CECT 434 (*E. coli* B+), *Ligilactobacillus salivarius* CECT 4063 (*L. salivarius*), *Streptococcus mutans* CECT 479 (*S. mutans*), and *Streptococcus sanguinis* CECT 480 (*S. sanguinis*). The bacteria were cultured in Luria–Bertani (LB) broth for 24 h and then an inoculum 0.5 of the McFarland scale was prepared. Finally, bacteria were seeded by exhaustion on LB agar plates. Visual evaluation was performed daily for 5 days to monitor possible changes in growth. 10 mg of foam sample was used to prepare each compression molded pellet (2-ton pressure, 2 min, 25 °C). To demonstrate bacterial sensitivity, a commercial nalidixic acid (NA) disc loaded with 30 μg of the antibiotic was used as a control (Becton Dickinson and Co., BD BBL).

Release experiments were made with samples weighing around 15 mg. Each sample was incubated at 37 °C in a rotatory shaker at 80 rpm in a 2 mL microtube filled with 1 mL of the release medium for 1 week. Specifically, a phosphate buffer saline (PBS) supplemented with 70% ethanol was used (PBS : EtOH, 30 v : 70 v). At predetermined time intervals, the microtube was centrifuged at 5000*g* for 5 min, and the supernatant was collected, and 1 mL of fresh medium was added to the microtube to continue the drug release. Finally, samples were dissolved in 200 μL of chloroform and extracted with ethanol for curcumin and water for PHMB to recover the occluded drug. Drug concentrations were determined by UV spectroscopy using a Shimadzu 3600 spectrometer. All drug release experiments were carried out using three replicates, and the results were averaged.

## 3. Results and discussion

### 3.1. Spectroscopy

**Fig. 4:**
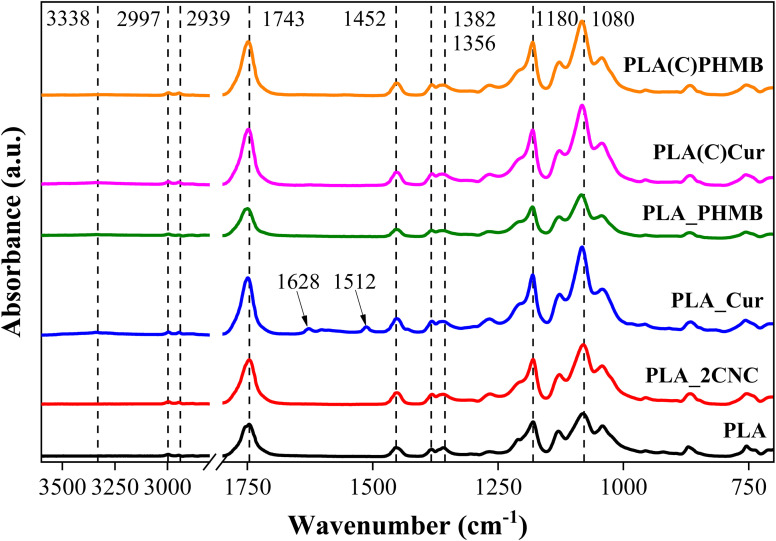
FTIR spectra of the samples with characteristic peaks.

Fig. 4 presents the FTIR spectra of prepared PLA-based film and foam samples. The characteristic peaks of PLA are identified at 2997 and 2939 cm^−1^, corresponding to the asymmetrical and symmetrical stretching of the C–H bonds.^22^ The peak at 1743 cm^−1^ indicates the stretching of the carbonyl C=O bond from the ester linkage.^23^ Additionally, the bending of –CH_3_ is observed at 1452 cm^−1^, while the asymmetrical and symmetrical bending of –CH are present at 1382 and 1356 cm^−1^, respectively.^22^ Prominent peaks at 1180 and 1080 cm^−1^ are attributed to the asymmetrical stretching of the C–O bond in the ester.^24^

The PLA_Cur sample exhibits additional peaks; notably, the bands at 1512 and 1628 cm^−1^ are attributed to aromatic ring C=C stretching.^25^ The weight ratio of drugs and CNC to PLA is relatively small, making it challenging to locate pronounced peaks, such as those in the PLA_Cur sample, due to the sensitivity of the device. Some peak shifts, observable in the C=O and C–O bands, can be attributed to different chain arrangements (crystallization process) of PLA for foams and films (PLA and PLA_2CNC). Although the selected PLA grade is amorphous, the interactions of solvents and molecular chains can alter the structure during electrospinning compared to compression molding.^26^ For the foam samples, a slight presence of water is noted by the hydroxyl (O–H) stretching at 3338 cm^−1^, which is not observed in the films due to the additional heating during the compression molding stage.

### 3.2. Thermal properties

**Fig. 5:**
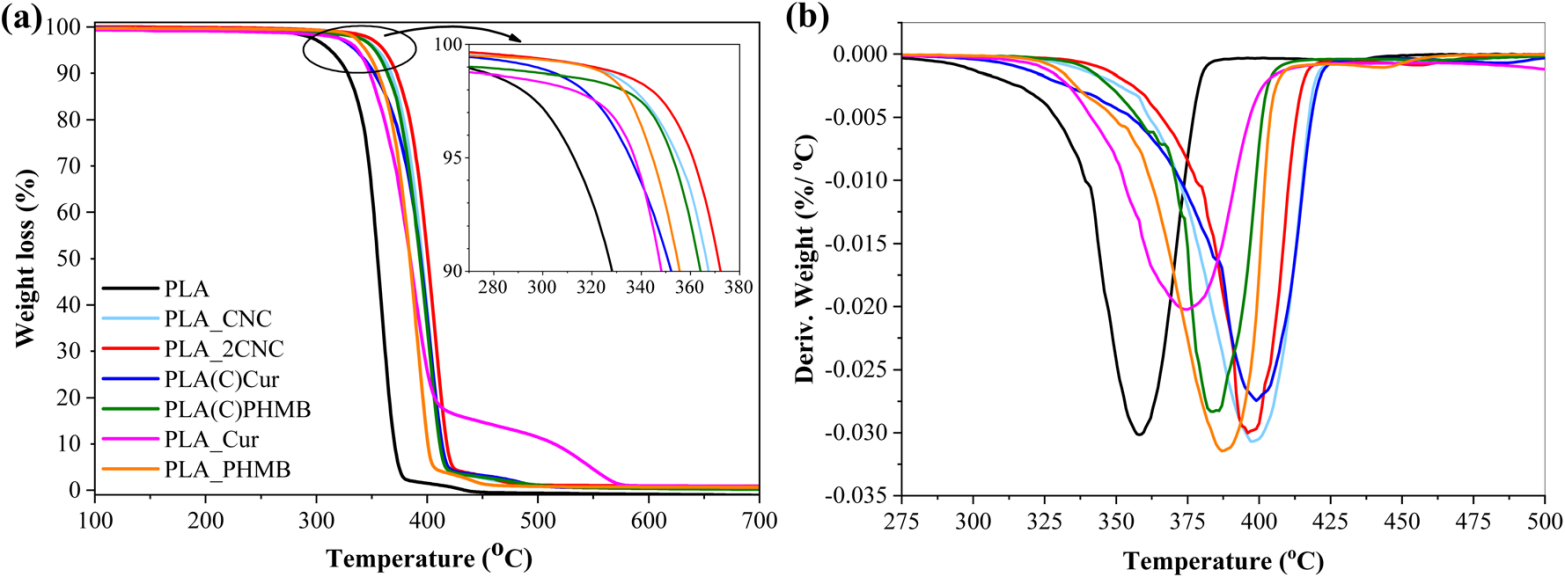
TGA curves: (a) the temperature evolution of weight loss and (b) its first derivative.

Fig. 5 shows the thermal degradation of PLA films and foams. Table 2 provides an overview of the thermal stability parameters: *T*_5%_, representing the temperature corresponding to 5% weight loss (which also marks the onset of degradation); *T*_max_, indicating the temperature at which the material undergoes the most significant weight loss; and the char yield at 700 °C. The first derivative curves (DTG) show only one decomposition stage but with a notable shift in the degradation peak (*T*_max_) to a higher temperature (up to 41 °C) compared to neat PLA, which exhibited a maximum degradation temperature of 358 °C. This shift to higher temperatures is attributed to CNC coating acting as a protective layer for PLA oxidation and subsequent degradation in the atmospheric conditions. Fig. 5a shows that initial degradation starts above 300 °C. Compositions containing Cur exhibited slightly faster degradation than other samples, whereas compression-molded films with CNC demonstrated the highest thermal degradation resistance. The final degradation step of PLA_Cur is slightly different from other compositions and can be explained with char formation on the surface of the sample, which slightly delays the degradation process. Overall, it is evident that the incorporation of CNC enhances thermal stability. Moreover, char yield was observed exclusively in samples containing CNC, indicating that CNC-derived char contributes to enhanced thermal stability.

**Table 2:** Thermal properties of PLA and PLA/CNC composites

Samples	*T*_m_, °C	Δ*H*_m_, J g^−1^	*T*_g_, °C	*T*_cc_, °C	*T*_5%_, °C	*T*_max_, °C	Char_700_, %
PLA	156.2	5.68	57.6^a^	99.2	313	358	0
			63.8^b^				
PLA_CNC	156.5	9.85	57.7^a^	126.6	355	398	0.7
			63.6^b^				
PLA_2CNC	154.3	15.54	57.9^a^	104.6	361	397	0.9
			59.6^b^				
PLA_Cur	—	—	—	—	337	375	0.8
PLA_PHMB	—	—	—	—	344	388	0.6
PLA(C)Cur	—	—	—	—	336	399	0.4
PLA(C)PHMB	—	—	—	—	337	384	0.2

a *T*_g_ from the cooling scans.

b *T*_g_ from the heating scans.

**Fig. 6:**
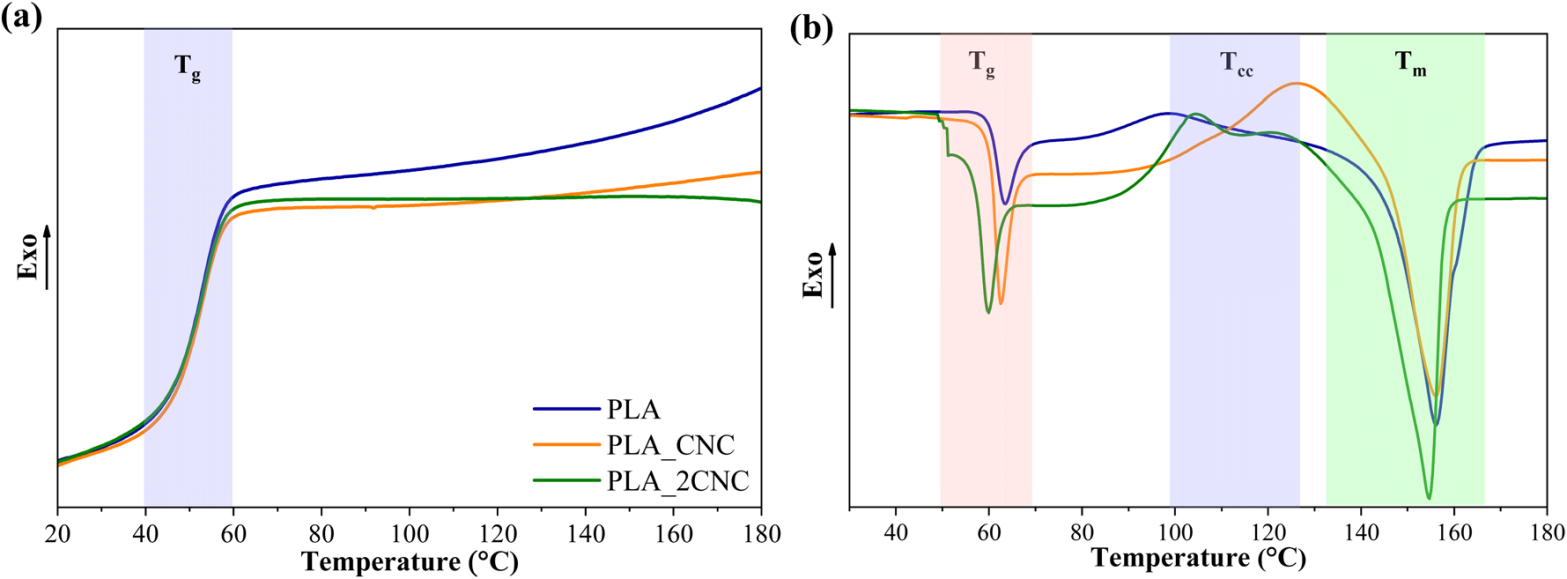
DSC (a) cooling and (b) second heating scans for neat PLA and PLA/CNC composites.

DSC was used to analyze the thermal properties of PLA and PLA/CNC composite films. Fig. 6 shows neat PLA and PLA/CNC cooling and second heating scans. Table 2 displays the calorimetric characteristics (melting temperature (*T*_m_), cold crystallization temperature (*T*_cc_), melting enthalpy (Δ*H*_m_), and glass transition temperature (*T*_g_) of the samples.

The cooling scans showed PLA glass transition temperature at about 57 °C for neat PLA and its composites. Also, exothermic crystallization peaks for neat PLA and PLC/CNC composites were not observed during cooling. PLA crystallization is relatively slow without nucleating fillers. In addition, the manufacturer specifies that the used PLA grade is amorphous.

The cold crystallization process is characteristic of PLA, with *T*_cc_ occurring at approximately 98 °C. The addition of 1 wt% CNC increased *T*_cc_ by about 30 °C, whereas 2 wt% CNC led to a smaller increase of only 9 °C. Literature reports indicate that CNC promotes nucleation but generally reduces the crystallization rate as its concentration increases.^27,28^ A higher CNC content may lead to the formation of larger aggregates, which are less effective in promoting nucleation.

The *T*_m_ of neat PLA is approximately 156 °C, with a *T*_g_ of about 64 °C. Adding 1 wt% CNC had no significant effect on these values. However, at higher CNC concentrations, both *T*_g_ and *T*_m_ decreased slightly—by about 5 °C and 2 °C, respectively. This reduction in thermal transitions is attributed to weakened intermolecular bonding between PLA chains. CNC aggregation at the interface disrupts PLA chain interactions and increases intermolecular spacing, enhancing chain mobility.^29^

### 3.3. Thermomechanical and mechanical analysis

**Fig. 7:**
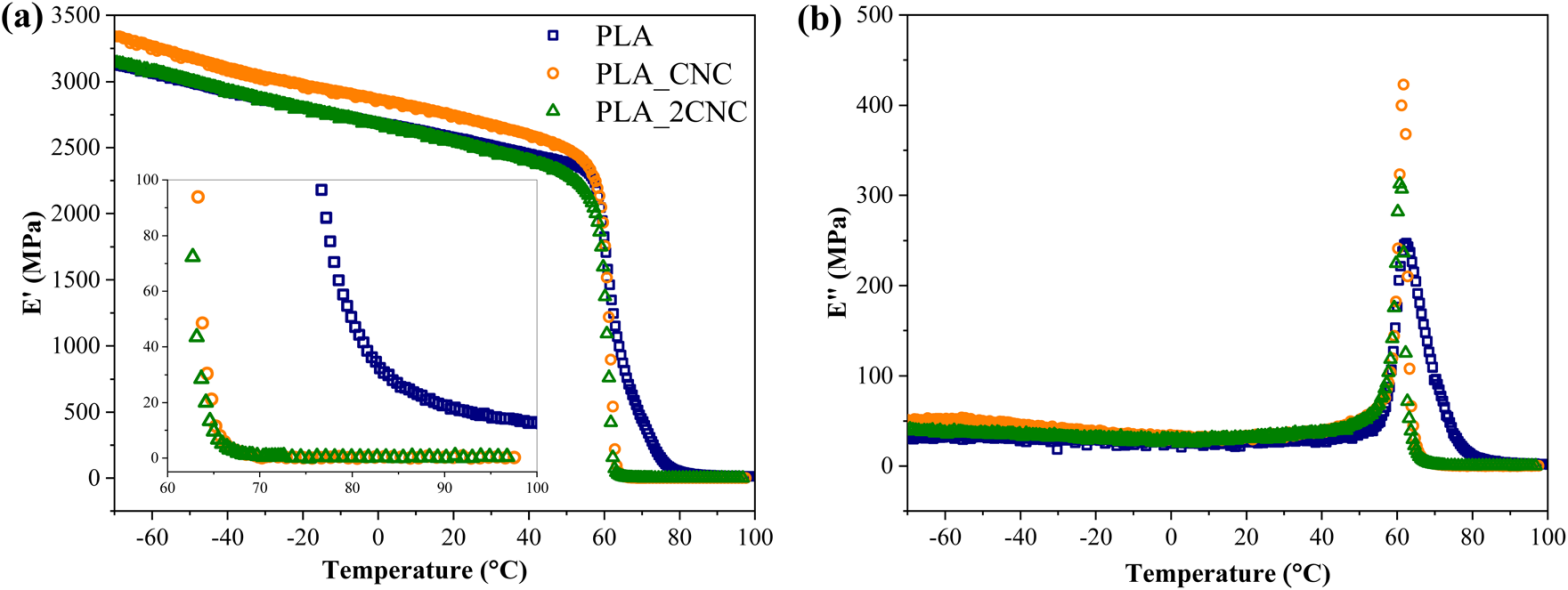
(a) Storage modulus (*E*′) and (b) loss modulus (*E*′′) of PLA/CNC samples.

The behavior of storage modulus (*E*′) and loss modulus (*E*′′) as a function of temperature for PLA and PLA/CNC nanocomposites containing 1% and 2% wt% of CNC are shown in Fig. 7. During compression molding of foams, CNC from the foam surfaces disperses into the PLA matrix. PLA_CNC and PLA_2CNC illustrate the dispersion of CNC and its influence on mechanical properties. In the glassy state (Fig. 7a), PLA_CNC demonstrates an increased storage modulus. However, PLA_2CNC exhibits properties similar to neat PLA, likely due to the formation of CNC agglomerates, which diminish reinforcement efficiency. Neat PLA shows a slightly delayed glass transition, resulting in higher storage modulus values between 60 and 80 °C. The sharper transition observed in samples with CNC suggests that CNC disrupts intermolecular bonding between PLA chains. Similarly, the loss modulus peak (Fig. 7b) indicates a reduction in glass transition temperature by up to 2 °C in CNC-containing samples compared to neat PLA (62 °C).

**Fig. 8:**
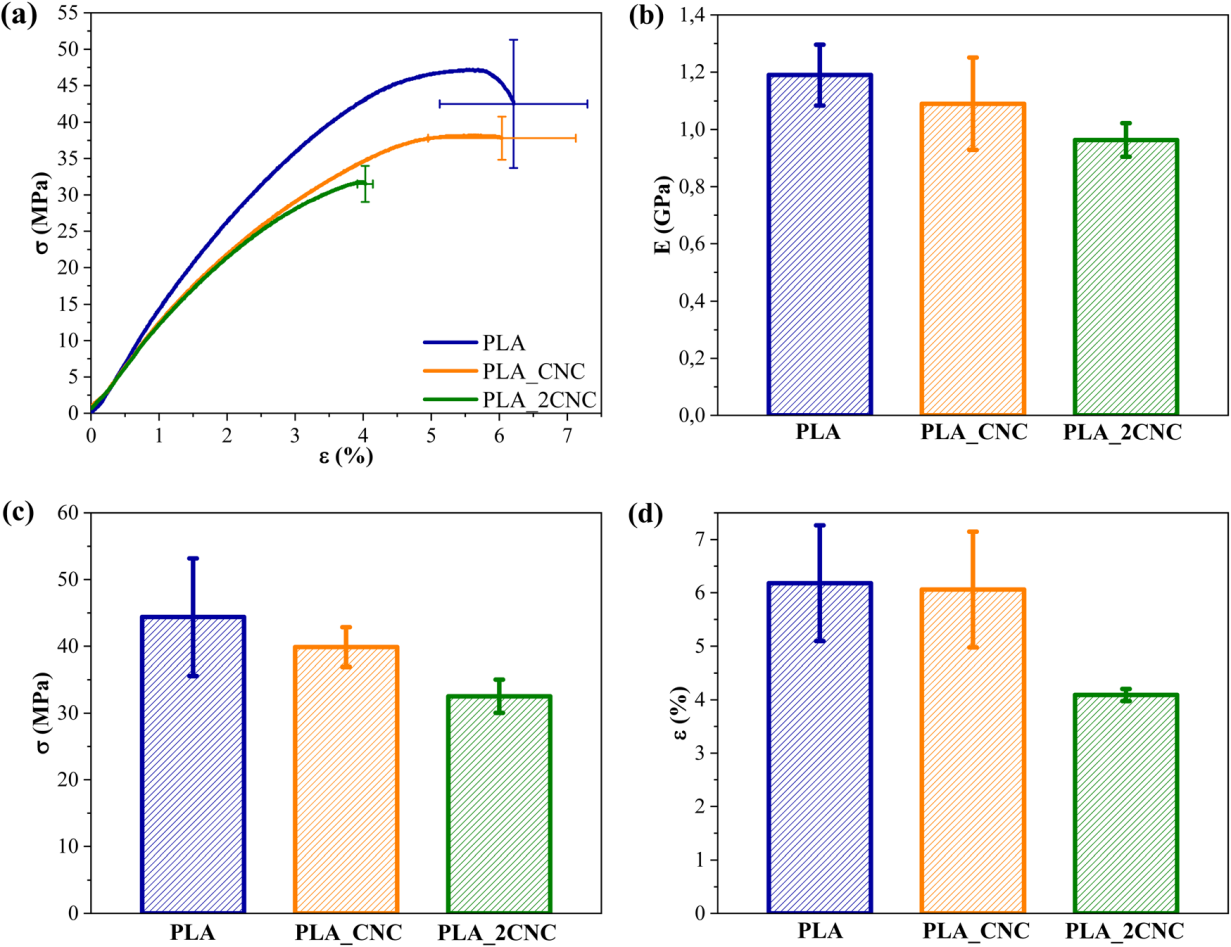
(a) Strain–strength curves, (b) elastic modulus (*E*), (c) tensile strength (*σ*), and (d) elongation at break (*ε*) for PLA and PLA/CNC films.

The representative stress–strain curves of the neat PLA and PLA/CNC nanocomposite films are presented in Fig. 8a. All curves show failure shortly after the elastic region, with minimal plastic deformation. The average values of elastic modulus (*E*), tensile strength (*σ*), and elongation at break (*ε*) are provided in Fig. 8b–d. The elastic modulus of neat PLA was 1.19 GPa. The addition of 1 wt% and 2 wt% of the cellulose filler to the PLA matrix caused a slight decrease in *E* values to 1.09 GPa and 0.96 GPa, respectively. The reduction in the elastic modulus can be attributed to the poor distribution of nanofiller. PLA's tensile strength (*σ*) gradually decreases after incorporating CNCs. The *σ* for neat PLA was 44.37 MPa, while PLA_CNC and PLA_2CNC demonstrated a decrease in *σ* values of 1.11- and 1.36-fold, respectively. The reduction in the tensile strength can be attributed to the agglomerates of the CNC within the composite structure, which resulted in local stress concentrations. The PLA achieved an elongation at break of about 6.2%. Strain at break (*ε*) is virtually unchanged for PLA_CNC compared to PLA. The PLA_2CNC composite, which contained 2 wt% of CNC, saw a notable drop to 4.09%.

The decrease in mechanical properties and toughness of composites could be associated with an aggregation of nanofillers. CNC agglomeration can act as stress concentrators, promoting defect propagation. These defects can grow to sizes more significant than the critical crack size, resulting in film failure.^30^

### 3.4. Antibacterial properties

**Fig. 9:**
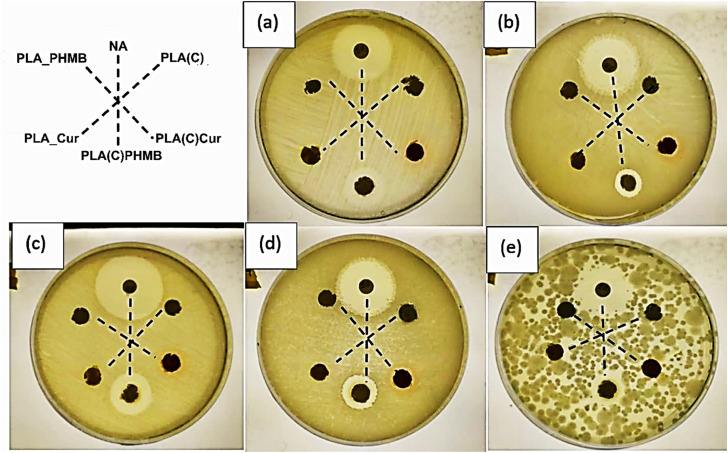
Agar diffusion test images at 24 hours for composite samples: (a) *E. coli* B+, (b) *L. salivarius*, (c) *E. coli*, (d) *S. mutans*, and (e) *S. sanguinis*.

The disc diffusion test, shown in Fig. 9, was used to evaluate the effectiveness of the antibacterial agents (Cur and PHMB) diffusion from the composite structure against five selected bacteria strains. The plates were evaluated every day for 5 days, but no changes were observed from the first day of evaluation. The film sample preparation route was not tested due to poor CNC dispersion and resulting heterogeneity. During incubation, the antibacterial agents diffuse radially outward from the discs through the agar. If the antibacterial agent is effective, it will inhibit bacterial growth, creating a clear, circular area around the disc known as the “zone of inhibition or halo of inhibition”. The concentration of the antibacterial agent is directly proportional to the area of the halo. Nalidixic acid (NA), as a broad-spectrum antibiotic, was used as a positive control to demonstrate bacterial sensitivity. It can be clearly seen that sample PLA(C)PHMB forms a visible continuing zone of inhibition in all tested bacterial media. Sample PLA(C)Cur exhibits good diffusion of Cur, as indicated by the distinct orange coloration, although it did not inhibit bacterial growth. Although it is often indicated that curcumin is an antibacterial agent, its correct activity is bacteriostatic; that is, even without producing bacterial death, it can slow bacterial growth. The results clearly reflect those compositions that used a coating approach and had successful diffusion of antibacterial agents compared to ones that integrated them into the electrospun fiber structure. The failure of PLA_PHMB and PLA_Cur to release antimicrobial agents from the structure could be attributed to the relative stability and slow biodegradation of PLA.^31^ The long PLA chains with strong intra- and intermolecular bonding significantly restrict the diffusion process. Furthermore, the inherently hydrophilic nature of cellulose, attributed to its abundance of hydroxyl groups, contrasts sharply with the predominantly hydrophobic molecular structure of PLA.

### 3.5. Drug release

**Fig. 10:**
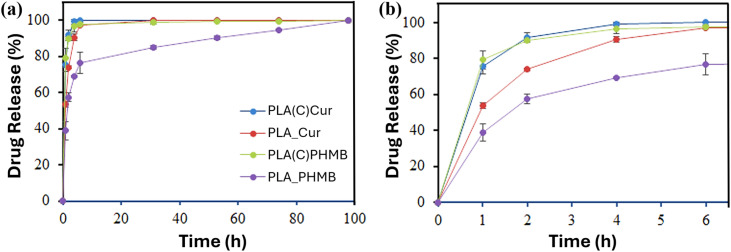
Drug release: (a) long-term release of Cur and PHMB over 98 hours and (b) initial release of the drugs.

Drug release experiments were conducted to elucidate the diffusion kinetics of the drugs from both the fiber-encapsulated and coating-encapsulated formulations (Fig. 10). A release medium composed of a 3 : 7 (v/v) mixture of PBS buffer and ethanol, chosen for its polarity to mimic blood serum^32^ and its suitability for spectrophotometric quantification, was used to evaluate drug release. As shown in Fig. 10a, all encapsulated drugs were released rapidly under these conditions. However, distinct differences in the initial release rates were evident (Fig. 10b).

Both Cur and PHMB in the coating exhibited a nearly complete release within 4 hours, whereas Cur encapsulated in PLA fibers (PLA_Cur) followed a similar trend but at a slower rate. The lowest release rate was observed for PHMB encapsulated in PLA fibers (PLA_PHMB). These findings suggest that the hydroxyl-rich CNC matrix facilitates rapid drug release, while the hydrophobic PLA matrix hinders diffusion. The hydrophilicity and size of the drug molecule also play a role in explaining the sustained release over 98 hours for PLA_PHMB.

The release behavior is further explained by kinetic constants derived from the Higuchi^33,34^ and first-order^35,36^ models, which together account for the overall drug release (Table 3). Finally, these results are consistent with the disc diffusion test and the observed antimicrobial and bacteriostatic effects of PHMB and Cur released from the coating. This also explains why such effects were not evident when the drugs were encapsulated in PLA fibers, as their reduced diffusion rates limited their release. In addition, it should be noted that the disc diffusion test used compression-molded pellets, which reduces the available surface area for drug release.

**Table 3:** Higuchi and first-order release constants using the combined model for drug release. *k*_H_ quantifies the drug release rate in the initial phase of delivery (0–60%) and *k*_1_ reflects the ability to reach the final equilibrium condition (40–100%)

Sample	Higuchi model^a^		First-order model^b^	
	*k*_H_ (h^−0.5^)	*r* (%)	*k*_1_ (h^−1^)	*r* (%)
PLA_PHMB	0.220	98.60	0.343	99.50
PLA-Cur	0.443	99.32	0.584	93.07
PLA(C)PHMB	0.687	99.43	0.809	96.40
PLA(C)Cur	0.656	98.57	0.842	99.91

a
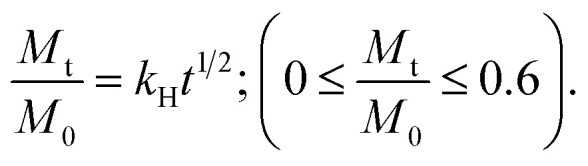

b
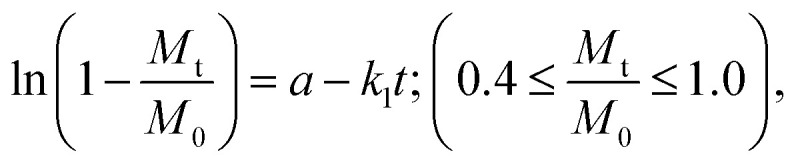
 where: *k*_H_ and *k*_1_ represent, respectively, the Higuchi and the first-order release constant; a is related to the release in the first step; *M*_t_ is the drug percentage released at time *t*; and *M*_0_ is the maximum drug percentage that can be released in the medium (*i.e.*, the total amount of drug).

## 4. Conclusions

We developed three distinct strategies for incorporating antimicrobial agents into a PLA matrix: direct encapsulation within electrospun fibers, within the CNC surface coating, and compression-molded films. The integration of CNC played a crucial role in enhancing both the hydrophilicity of PLA and the diffusion of antimicrobial agents, ultimately influencing drug release kinetics. Thermogravimetric analysis confirmed enhanced thermal stability in all CNC-modified samples. However, CNC dispersion was poor in compression-molded films, leading to heterogeneity and limiting their applicability.

Agar diffusion tests revealed that drug release behavior depended strongly on the incorporation method. Drug-coated fibers enabled rapid and efficient diffusion, particularly for PHMB, which exhibited strong antibacterial effects. In contrast, Cur displayed primarily bacteriostatic properties. Drug release in a 3 : 7 (v/v) PBS–ethanol medium, chosen to mimic blood serum polarity, showed similar initial kinetics for both fiber-embedded and surface-coated drugs. Drug-coated fibers released most of the drug within four hours, while encapsulated PHMB and Cur achieved 68% and 90% release, respectively. Notably, PLA_PHMB exhibited sustained drug release over 98 hours. Hydrophilicity and drug molecule size contributed to the extended release observed for PLA_PHMB.

This study highlights the potential of CNC-based surface modification as an effective approach for tailoring the release profile of drugs in PLA-based materials. Embedding antimicrobial agents within electrospun fibers may be beneficial for sustained and controlled drug delivery applications, while surface coating strategies offer a more immediate and effective response. Future research should focus on optimizing the matrix composition and tuning the release kinetics to expand biomedical applications.

## Data availability

The data for this article have been included in the main article and can be read from curves or images presented in the figures.

## Conflicts of interest

The authors declare that they have no known competing financial interests or personal relationships that could have appeared to influence the work reported in this paper.
